# Marine protected areas rescue a sexually selected trait in European lobster

**DOI:** 10.1111/eva.12992

**Published:** 2020-05-22

**Authors:** Tonje Knutsen Sørdalen, Kim Tallaksen Halvorsen, Leif Asbjørn Vøllestad, Even Moland, Esben Moland Olsen

**Affiliations:** ^1^ Department of Natural Sciences Centre for Coastal Research University of Agder Kristiansand Norway; ^2^ Institute of Marine Research Flødevigen Norway; ^3^ Department of Biosciences Centre for Ecological and Evolutionary Synthesis (CEES) University of Oslo Oslo Norway

**Keywords:** claws, fisheries‐induced evolution, *Homarus gammarus*, marine reserves, secondary sexual trait, selective harvesting, sexual selection, trap fisheries

## Abstract

Marine protected areas (MPAs) are increasingly implemented worldwide to maintain and restore depleted populations. However, despite our knowledge on the myriad of positive responses to protection, there are few empirical studies on the ability to conserve species’ mating patterns and secondary sexual traits. In male European lobsters (*Homarus gammarus*), the size of claws relative to body size correlates positively with male mating success and is presumably under sexual selection. At the same time, an intensive trap fishery exerts selection against large claws in males. MPAs could therefore be expected to resolve these conflicting selective pressures and preserve males with large claws. We explored this hypothesis by contrasting claw size of males and females in three pairs of MPAs and nearby fished areas in southern Norway. By finding that male lobsters have up to 8% larger claws inside MPAs compared to similarly sized males in fished areas, our study provides evidence that MPAs rescue a secondary sexual trait. Recovery from harvest selection acting on claws is the most likely explanation; however, the higher abundance of lobster inside MPAs does not rule out a plastic response on claw size due to increased competition. Regardless of the underlying cause, our study demonstrates (a) the value of protected areas as a management tool for mitigating fisheries‐induced evolution and (b) that MPAs help maintaining the scope for sexual selection in populations with vulnerable life histories and complex mating system.

## 1 INTRODUCTION

Natural and sexual selection are the fundamental evolutionary processes shaping the traits of species and populations. Yet, humans can act as a very strong selective agent sometimes opposing what is favoured by natural selection pressures (Carlson et al., 2007). Phenotypic shifts induced by selective hunting and fishing have ecological consequences and can drive evolution within contemporary timescales (Conner, 2003; Darimont et al., 2009; Palkovacs, Moritsch, Contolini, & Pelletier, 2018). Typically, commercial harvesting targets the larger and more valuable individuals, whereas recreational harvesting and poaching in addition may target some animals with conspicuous ornaments or weaponry, such as horns, antlers and claws (Chiyo, Obanda, & Korir, 2015; Coltman et al., 2003; Oliveira et al., 2000). In large terrestrial animals subjected to trophy hunting or ivory trade, selective harvest of primarily superior and sexually dominant males has been shown to induce artificial selection and hence evolution towards smaller horn size, reduction in male body size or, in the case of elephants, loss of tusks (Chiyo et al., 2015; Coltman et al., 2003; Martin, Festa‐Bianchet, Coltman, & Pelletier, 2016; Pigeon, Festa‐Bianchet, Coltman, & Pelletier, 2016). Such conspicuous traits are fundamental to the outcome of competitive interactions and are the results of strong sexual selection (Swain, Sinclair, & Mark Hanson, 2007; Wilber, 1989; Woolmer, Woo, & Bayes, 2013).

Well‐functioning mating systems are perceived as the foundation for population resilience and growth rate (Allendorf & Hard, 2009). Recent evidences suggest that reducing the opportunity for sexual selection by removing individuals with higher expression of secondary sexual traits can lower population fitness and increase the extinction risk under environmental change (Cally, Stuart‐Fox, & Holman, 2019; Knell & Martínez‐Ruiz, 2017; Lumley et al., 2015; Plesnar‐Bielak, Skrzynecka, Prokop, & Radwan, 2012). This is because secondary sexual traits are likely to be honest signals of “good genes” reflecting the owner's overall genetic match to the environment (Weatherhead & Robertson, 1979). If the environment changes, the best adapted individuals should afford the highest expression of secondary sexual traits and thus gain mating success (Lorch, Proulx, Rowe, & Day, 2003; Siller, 2001; Whitlock & Agrawal, 2009). Consequently, sexual selection can improve population mean fitness and be able to drive adaptation at a much higher rate than natural selection alone (Lorch et al., 2003; Lumley et al., 2015).

Nature reserves, or protected areas, should have the potential to restore sources of individuals that are not affected by harvest selection. In trophy‐hunted bighorn rams (*Ovis canadensis*), individuals harvested near protected areas in Canada had larger average horn size compared to rams shot far from protected areas (Pelletier, Festa‐bianchet, Jorgenson, Feder, & Hubbs, 2014), and in Zimbabwe, horn size of impala (*Aepyceros melampus*) decreased with distance from a national park (Crosmary et al., 2013). In oceans and coastal areas worldwide, marine protected areas (MPAs) are increasingly being implemented to restore depleted populations, improve ecosystem health and benefit fisheries through spillover effects (Hastings & Botsford, 2003; Pendleton et al., 2018). Although there is mounting evidence of how number, biomass, size and age of fish and invertebrate species within MPAs are often much greater than in comparable areas open to fishing (Baskett & Barnett, 2015; Gillespie & Vincent, 2019; Halpern, 2003; Lester et al., 2009; Russ, Cheal, & Dolman, 2006), it is rare to assess the potential for MPAs to preserve or restore secondary sexual traits. Thus, this warrants further investigation since secondary selected traits are likely affected by fishing, especially in the light of many recent studies demonstrating harvest selection on behavioural or morphological traits independently of body size (Alós, Palmer, Linde‐Medina, & Arlinghaus, 2014; Arlinghaus et al., 2017; Biro & Sampson, 2015). In salmonid fishes, secondary sexual traits (body depth) have also been shown to correlate with increased catchability, which may affect the opportunity and strength of sexual selection (Hamon & Foote, 2005; Kendall & Quinn, 2013).

The secondary sexual traits of many harvested fish species may be cryptic and sometimes poorly described or identified. However, some commercially important crustaceans have dimorphic chelae—or claws in adults with a major molar‐toothed (crusher) claw and a minor incisor‐toothed (cutter) claw. In most species, males grow larger and heavier claws than females and are considered secondary sexual traits (Hartnoll, 1974; Mariappan, Balasundaram, & Schmitz, 2000; Stein, 1976; Templeman, 1935). The claws are tools used in foraging and in defence against predators, but are also weapons used in male–male conflicts (armaments) and signals indicating fighting ability and attractiveness towards females (ornaments) (Atema, 1986; Elner & Campbell, 1981; Jivoff, 1997; Sneddon et al., 1997). A recent field study on European lobster (*Homarus gammarus*) found that large claws increase male mating success. Specifically, sexual selection seems to be acting more strongly on relative claw size (with respect to body size) than on absolute claw size or body size (Sørdalen et al., 2018). Furthermore, the strength of sexual selection in males appeared to be higher inside a marine protected area relative to a nearby, heavily fished area (Sørdalen et al., 2018). A telemetry study conducted in the same fished area found that relative claw size was positively correlated with capture probability and hence mortality in the trap fishery (Moland, Carlson, Villegas‐Rios, Wiig, & Olsen, 2019). This means that harvest selection against large claws may become effective as soon as lobsters reach the minimum size limit in the fishery. Thus, both sexual selection and harvest selection have been identified to act on the same trait, but in opposite directions. Hence, MPAs should be able to preserve males with large claw phenotypes, assuming any genetic component underlying claw size is not strongly reduced by past fishing. The effect on females is expected to be smaller; harvest selection has not been studied in female lobsters but is presumably weaker than on males because all egg‐bearing females are protected, and they have lower catchability than males (Moland, Ulmestrand, Olsen, & Stenseth, 2013). In this study, we address these hypotheses by comparing the relationship between body and claw size of lobsters inside and outside three lobster reserves established in 2006. By confirming our prediction that lobsters, particularly males, have larger claws relative to body sizes inside protected areas, this study documents the usefulness of MPAs to preserve a trait under sexual selection.

## 2 MATERIALS AND METHODS

### 2.1 Species and study system

**FIGURE 1:**
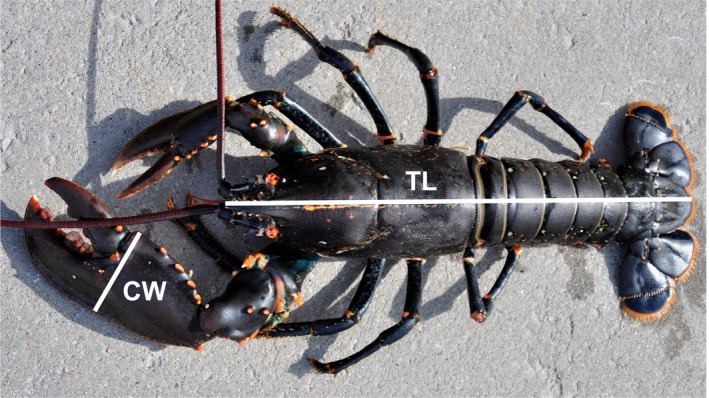
Study species. European lobster (*Homarus gammarus*) (male) showing positions of measurement: body size; total length (*TL*); claw size; and crusher claw width (*CW*)

European lobster (hereafter, lobster) are large, long‐lived sexually dimorphic crustaceans in temperate waters distributed from the north of Norway to Morocco in North Africa, including the Mediterranean Sea (Triantafyllidis et al., 2005). Males grow faster, mature at smaller size and have relatively larger greater chelae (hereafter, claws) than females (Debuse, Addison, & Reynolds, 2001; Lizárraga‐Cubedo, Tuck, Bailey, Pierce, & Kinnear, 2003). The average age of large (150–170 mm carapace length, *CL,* measured from rear of the eye socket to the rear of the carapace) males and females is estimated to be 31 and 54 years, respectively (Sheehy, Bannister, Wickins, & Shelton, 1999). One of the largest specimens was estimated to be 650 mm (total length, *TL,* measured from tip of rostrum to mid‐tail; Figure 1) based on recovery of a large crusher claw (360–370 mm long) in Skagen, Denmark (Wolff, 1978). The lobster is one of the most valuable and sought‐after species in Northern Europe's commercial and recreational fisheries. In Norway, the lobster catch rates have declined by 65% from the 1950s to 2000s and is today at the lowest record in history with no sign of recovery (Pettersen, Moland, Olsen, & Knutsen, 2009). In response, the fishery is now mostly recreational and strictly managed by gear restrictions (10 and 100 traps for recreational and commercial fishers, respectively), slot size restrictions and an open season from 1 October to 30 November. A ban on the harvest of egg‐bearing females was implemented in 2008, along with an increase in minimum legal size to 250 mm total length. In 2017, a maximum size limit at 320 mm *TL* was introduced for lobster caught along the Norwegian Skagerrak coastline (Sørdalen et al., 2018).

**FIGURE 2:**
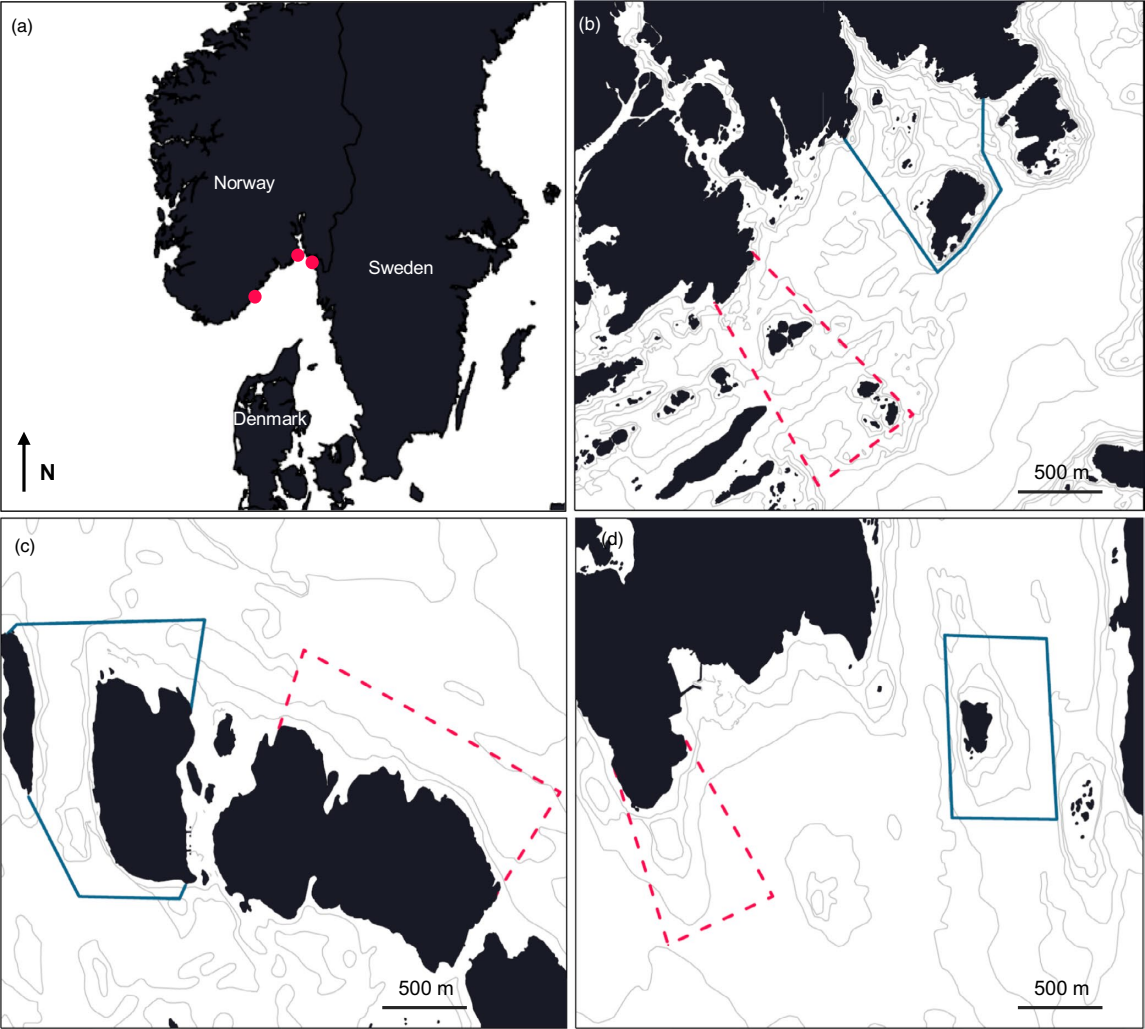
Sampling location. From top, (a) red circles show the location of study areas in three counties on the Norwegian Skagerrak coast and reads from left to right; (b) Aust‐Agder, (c) Vestfold and (d) Østfold. The lobster MPAs are represented by solid blue lines and fished control areas by broken red lines

Three small‐scale lobster reserves located off the Skagerrak coast were established in September 2006 with the primary goal to investigate the potential for rebuilding local populations and to assess the effects of fishing. The three MPAs, Flødevigen in Aust‐Agder County (~1 km^2^, 58°25′N, 8°45′E), Kvernskjær in Østfold County (0.5 km^2^, 59°02′N, 10°58′E) and Bolærne in Vestfold County (~0.7 km^2^, 59°13′N, 10°31′E; Figure 2), have regulations that prohibit any capture of lobster and ban the use of passive fishing gears such as fyke nets and traps. Harvesting of marine resources is only permitted using hook and line and with rules defined by the Norwegian Directorate of Fisheries. To establish a baseline in each county and enable scientific monitoring of the effects of protection over time, each of the reserves is accompanied by monitored unprotected areas where lobster fishing is allowed in accordance with current management regulations. These adjoining areas cover approximately the same size and habitat composition (kelp and rocky bottom substrate) as the MPAs. The distance between each protected and fished area is 1.7, 0.9 and 2.3 km (from area centre) in Aust‐Agder, Østfold and Vestfold, respectively (Figure 2). Mark–recapture data suggest limited exchange of harvestable adult individuals between the fished areas and MPAs in any of the counties (Fernández‐Chacón et al., 2020; Thorbjørnsen et al., 2018).

### 2.2 Sampling design and lobster data

We sampled lobster as part of a standardized capture–mark–recapture sampling programme conducted annually by the Norwegian Institute of Marine Research (IMR) in all three counties. Claw data were collected between 2017 and 2019. Each of the MPAs and the monitored fished areas was sampled simultaneously before the start of the fishing season, between 20 August and 10 September each year, so that shared temporal effects can be accounted for. All lobster were caught using a set of 25 individually marked mackerel‐baited two‐chambered Parlour traps placed at the bottom at depths between 8 and 30 m, with a 24‐hr soak time prior to each haul (yielding 100 hauls in each area per year). The traps were positioned evenly throughout the study areas at preferred lobster habitats, typically rocky and hard bottom substrate. Traps used in the fishery have escape openings to allow undersized lobster to escape. The traps used in the sampling programme, however, have no escape openings to increase the size range of captured animals. Data on location, depth and lobster catch of every trap were recorded. All captured lobster were sexed, and total length (*TL*) was measured to the nearest millimetre as a measure of body size. We measured the width of the major crusher claw as the widest part of the crusher claw (across the “palm” beneath the top of the ridge of dactyl) to obtain a measure of claw size (*CW*; see Figure 1). Each lobster was individually tagged with externally visible T‐bar tags (TBA2, 45 × 2 mm; Hallprint) and released at the sampling site.

### 2.3 Statistical analyses

The catch‐per‐unit‐effort (CPUE) was plotted for a visual comparison of population density between reserves and fished areas over the last 13 years. To provide a measure of mean density for the years with claw measures, we averaged the CPUE over the last 3 years (2017, 2018 and 2019). To investigate the effects of protection on claws, we first tested whether the probability of missing one or both claws differed between areas (*Status*), adjusted for body size (*TL*). Injuries to and loss of appendages are common among decapod crustacean and are exacerbated by fishing methods (Juanes & Smith, 1995). General linear models were fitted separately for males and females with claw loss as the binomial response variable:

$$\text{Clawloss}∼\beta_{0}+\beta_{1}Status+\beta_{2}TL$$

Second, we used a linear model to test the prediction that male lobster in MPAs have larger claws than conspecifics in the contrasted fished areas. Again, we fitted the same model to the data on female lobsters. The following a priori‐defined general linear model structure was applied:

$$CW∼\beta_{0}+\beta_{1}TL+\beta_{2}Status+\beta_{3}County+\beta_{4}TL:Status$$

*CW* (crusher claw width) is the response variable, with the factor *County* accounting for spatial differences among the three counties (Aust‐Agder, Vestfold and Østfold) as levels. As a first step, we censored any lobsters that had very small crusher claws, most likely resulting from a regeneration of a lost claw. Claw loss inhibits growth in crustaceans (Moriyasu, Landsburg, Wade, & Maynard, 1999), and in the American lobster (*Homarus americanus*), regenerated claws are typically smaller than intact claws, even after multiple moults (Cheng & Chang, 1994). In order to distinguish lobster with regenerated claws from those with naturally small claws, we conducted the following analysis: we assumed the residuals from model 1 to be normally distributed with a mean of zero. We then sequentially removed the individual with the largest negative residual value and refitted the model until the largest negative residual was equal to or smaller than the largest positive residual value. This method of classifying lobster with regenerating claws would ensure that we are conservative in identifying individuals (with regenerating claws) that should be excluded in our final analysis (34 females and 19 males were thus excluded; see Figure S1). We focused our analysis on lobster larger than the minimum size limit (250 mm *TL*) since harvest selection is assumed to only operate on legal‐sized lobster (Fernández‐Chacón et al., 2020). Further, the fished areas had a truncated size distribution; of the 306 lobsters above the maximum legal size limit (320 mm *TL*), only 4.6% were caught in the fished areas. Thus, we restricted our models to compare only the overlapping size range between fished areas and MPAs (*TL*_max males_ = 362 mm, *TL*_max females_ = 355 mm; Table 1). The data and predictions from the model also including the large MPA lobsters are shown in Figure S2. We reran the models with the final dataset and focused on the model terms involving *Status* (MPAs or fished area). If harvest selection acts on relative claw size, a significant interaction effect can be expected due to cumulative selective mortality that should lead to increasing difference with age (which is assumed to be closely correlated with body size). The interaction was dropped if nonsignificant (*p* > .05). The three sampling years were pooled as a preliminary model revealed no year effect on claw width (results not shown). All statistical analyses were performed in R 3.5.1 (R Core Team, 2018).

**TABLE 1:** European lobster (*Homarus gammarus*)

Area	Aust‐Agder		Vestfold		Østfold	
Status	Flødevigen MPA	Fished	Kvernskjær MPA	Fished	Bolærne MPA	Fished
Males						
No. males	206	69	310	147	261	228
Mean total length (range), mm	288 (152–414)	237 (162–315)	283 (145–385)	252 (165–362)	279 (184–377)	245 (166–340)
Mean claw width (range), mm	58 (23–105)	42 (25–60)	58 (22–92)	47 (19–80)	57 (29–97)	46 (25–69)
Females						
No. females	227	70	318	129	385	210
Mean total length (range), mm	294 (164–395)	248 (147–337)	282 (183–395)	254 (153–355)	283 (143–424)	245 (156–340)
Mean claw width (range), mm	48 (25–70)	40 (23–54)	46 (26–66)	41 (24–59)	47 (22–73)	39 (22–53)

Note

Summary of individuals sampled in the annual research trap survey in 2017, 2018 and 2019, separated in protected (MPA) and fished areas of Aust‐Agder, Vestfold and Østfold. Number of males and females, mean body size (total length, *TL*) and mean claw size (crusher claw width, *CW*) in millimetres with size ranges. Data include regenerated claws. *N* = 2,560.

Abbreviation: MPA, Marine protected area.

## 3 RESULTS

**FIGURE 3:**
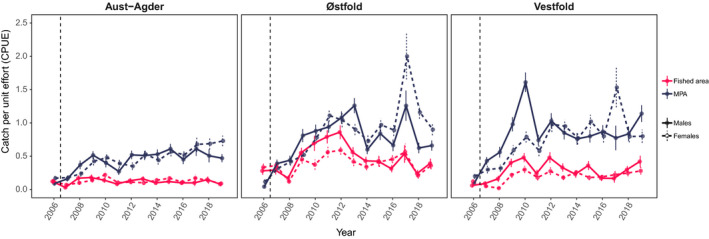
Mean catch distribution of adult lobster in the marine protected areas (MPAs) and fished areas. Populations are from the lobster MPAs (dark blue) and fished areas (red) of Aust‐Agder, Vestfold and Østfold during the 13 years of protection (2006–2019). Catch‐per‐unit‐effort (CPUE) is shown for lobsters above the legal size limit of 250 mm total length (*TL*) from the annual research trap survey. Implementation of the protected areas from September 2006 is indicated by vertical stippled line. Error bars depict one standard error around the mean. Sex is separated with males in solid line and females in broken line

All MPA populations have responded well to protection with notable increases in mean catch‐per‐unit‐effort (CPUE) for legal‐sized lobster. From 2017 to 2019, the same years as claw measurements were taken, and the lobster catches in the MPAs were 5.08 times higher in Aust‐Agder, 3.58 times higher in Vestfold and 2.87 times higher in Østfold than in their respective fished areas (Figure 3).

In total, 2,656 lobster were fished in the three counties in the scientific sampling surveys between 2017 and 2019. Lobsters with missing claw(s) accounted for 4.3% of the total catch, 5.4% in MPAs and 2.2% in fished areas. There was no relation between claw loss and body size in either males (*β* = 0.00003, *SE* = 0.0001, *p* = .82) or females (*β* = 0.0002, *SE* = 0.0001, *p* = .13). Moreover, females were more likely to miss a claw in the MPAs (*β* = 0.04, *SE* = 0.01, *p* = .01), but no such difference was evident for males (*β* = 0.01, *SE* = 0.01, *p* = .19). After removing individuals with missing claws and with incomplete measurement data, 2,560 lobster (1,339 females and 1,221 males) had intact crusher claws: 1,707 from the MPAs and 853 from the fished areas (Table 1). The proportion of lobster with regenerated claws was similar in the MPAs (3.83%) and the fished areas (3.35%) (see Figure S1). Lobster identified as having regenerated claws were then excluded from the following analysis.

**FIGURE 4:**
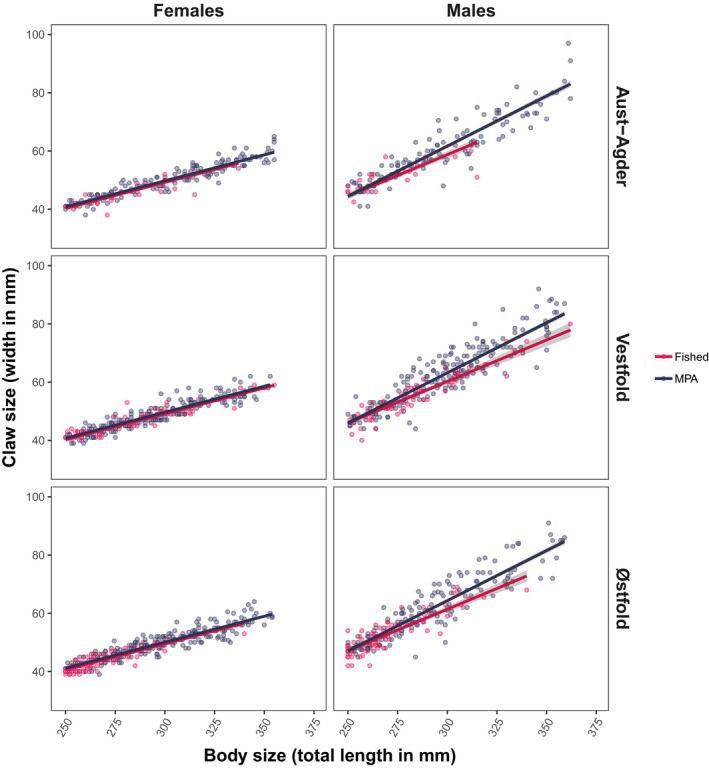
Claw sizes. Regression of claw size (claw width, *CW*, in mm) to body size (total length, *TL*, in mm) in female and male European lobster, sampled in lobster marine protected areas and fished areas of Aust‐Agder, Vestfold and Østfold in 2017–2019. The areas in grey are 95% confidence intervals. Number of observations *N* = 1,426

**FIGURE 5:**
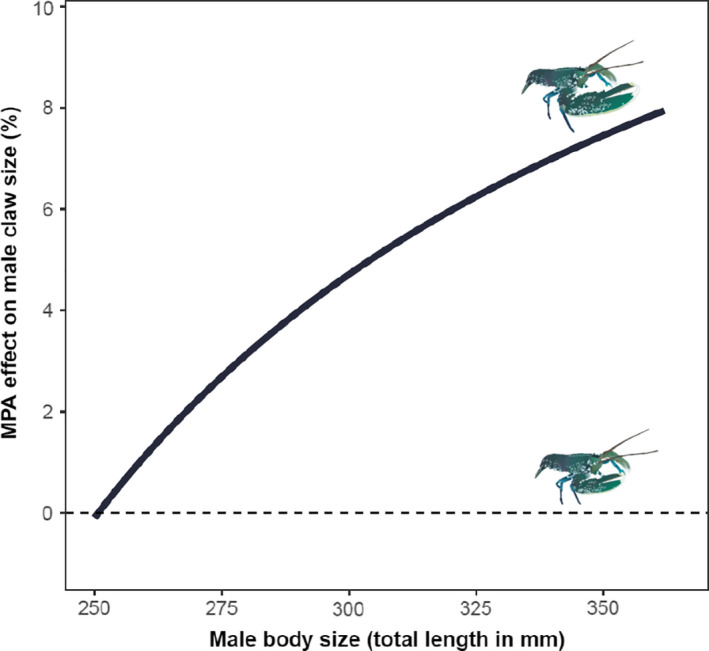
Marine protected area (MPA) effect on male claw size. The curves indicate the percentage difference in predicted male claw size (claw width, *CW*, in mm) in MPA relative to fished area in Vestfold County (see Table 2 for model summary). The upper limit of the curve corresponds to the largest male caught in fished area (362 mm total length, *TL*). Dashed line is where the claw size of males in MPA and fished area are of equal size, which is only at the minimum size limit (the MPA effect occur after 250 mm *TL*)

The size of claws increased more rapidly with increasing body size for males in the MPAs than in the fished area (Figure 4; Table 2; *TL* × *Status* interaction: *β* = 0.06, *SE* = 0.02, *p* < 0001). Using Vestfold as an example, the model predicted that the average male entering the fishery (250 mm *TL*) would have the same crusher claw size in the fished area and the protected area (0.1% smaller in the MPA). However, with increasing body size, all claws became progressively larger in the MPAs; claws were an estimated 8% larger in MPA males with a body size of 362 mm *TL*, which corresponds to the largest male captured in the fished area (Figures 4 and 5). After removing the nonsignificant interaction effects for females (*β* = 0.01, *SE* = 0.01, *p* = .18; complete model summary not shown), their relative claw size also differed between fished and protected areas as the additive effect of Status was significant (*β* = 0.56, *SE* = 0.17, *p* = .001; Figure 4, Table 2).

**TABLE 2:** Model estimations

	Explanatory variable	Estimate	*SE*	*t*‐Value	*p*
Males (*n* = 633) Multiple *R* ^2^ = .85	Intercept	−26.78	3.93	−6.82	**<.001**
	Total length	0.29	0.01	20.1	**<.001**
	Status—MPA	−14.85	4.4	−3.4	**.001**
	County—Vestfold	1.5	0.43	3.51	**<.001**
	County—Østfold	2.7	0.44	6.06	**<.001**
	Total length:Status—MPA	0.06	0.02	3.81	**<.001**
Females (*n* = 793) Multiple *R* ^2^ = .87	Intercept	−4.28	0.75	−5.7	**<.001**
	Total length	0.18	0.003	68.89	**<.001**
	Status—MPA	0.56	0.17	3.24	**.001**
	County—Vestfold	−0.09	0.18	−0.52	.607
	County—Østfold	0.31	0.18	1.72	.086

Note

Summary of the linear models between claw size (crusher claw width, *CW*, in mm) and body size (total length, *TL*, in mm) of male and female lobster. The fished area and the Aust‐Agder County are set as reference levels (ref. Table 1).

Significant values are indicated in bold.

Abbreviation: MPA, Marine protected area.

## 4 DISCUSSION

Here, we show that MPAs are home to lobster with larger claws compared to areas open to fishing. This finding is consistent with the prediction of population changes in the mean value of a sexual trait released from ongoing fisheries‐induced selection (Moland et al., 2019). Thus, this study demonstrates that MPAs can both rescue and promote a secondary sexual trait eroded by selective harvesting. We deem our finding to be a valuable contribution towards a broader understanding of the benefits of MPAs. Below, we discuss possible explanations of the observed patterns, the importance of preserving secondary sexual traits in harvested populations and the potential of marine reserves for restoring phenotypic trait variation, natural selection and mating patterns.

### 4.1 Claw size in relation to protection and abundance

The larger claws of lobster in the MPA are most likely a reflection of the high and selective fishing mortality outside MPAs (Fernández‐Chacón et al., 2020; Moland et al., 2019). Larger claws in males are known to indicate social dominance (Skog, 2009a) where even a small difference in claw size dictates victory in contests and a superior status in a hierarchy (Atema & Cobb, 1980; Van Der Meeren & Uksnøy, 2000). Locally dominant males will also successfully attract and mate with females in the wild (Karnofsky & Price, 1989), and it has previously been shown that claw size is a sexually selected trait in the same study populations (Sørdalen et al., 2018). Therefore, aggressive behaviour towards conspecifics and defence of resources, such as baited traps, have been suggested to be underlying mechanisms driving the fishery selection. Individual behavioural traits (boldness, aggression, activity, sociability) are increasingly being recognized as determinants of catchability in fisheries (Arlinghaus et al., 2017; Diaz Pauli & Sih, 2017). In crayfish (*Cherax destructor*), bolder individuals are more likely to be attracted to and captured in baited traps because they also grow faster and require more food (Biro & Sampson, 2015). Furthermore, correlations between boldness (i.e. the propensity to take risks) and strength in the expression of secondary sexual traits have been shown in some fish (Fabre, GarcÍa‐Galea, & Vinyoles, 2014; Godin & Dugatkin, 1996) and lizards (Putman, Azure, & Swierk, 2019). Thus, sexual selection may favour certain personality traits (e.g. boldness) associated with the achievement of strong expression of secondary sexual traits (Fabre et al., 2014), such that claw size may be correlated with dominant behaviour that increases the catchability. Lastly, passive gears will be selective to some extent and may therefore not representatively sample the populations we are studying, although we regard it as unlikely that capture selection related to morphology affected our results because we analysed a restricted size range (max 250–365 mm *TL*).

Since the implementation of the three lobster reserves in 2006, abundance and size composition in the areas have changed considerably (Fernández‐Chacón et al., 2020; Moland et al., 2013; Sørdalen et al., 2018; Figure 3 this study). Intraspecific competition may therefore be higher in MPAs, which can increase the fitness benefit of having larger claws. Thus, an alternative explanation for larger relative claw size in the MPAs could be that the increased density in the reserves induces males to invest in larger claws as a response to a more competitive environment. It is unclear how increased density may affect competition and interactions in wild European lobster. For example, experimental work has found that males suppress their dominance interactions when shelters are scarce, while females fight more often when shelters are abundant (Debuse, Addison, & Reynolds, 2003). In other crustaceans, the expression of claws has been shown to develop plastically in response to variation in diet (prey) and temperature (Baldridge & Smith, 2008; Edgell & Rochette, 2009; Smith, 2004; Smith & Palmer, 1994). The relative influence of plasticity or harvest selection on claw size in lobster is unknown, but density‐dependent phenotypic plasticity is most likely pulling in the same direction as harvest‐induced selection.

The results showed that relative claw size of females also differed between protected and fished areas, although the effect was much weaker compared to that of males. Moland et al. (2019) did not investigate whether the fishery is selective on female claw size, which would have been helpful in elucidating whether harvest selection is acting on the same traits in males and females, which our results indirectly suggest. Female lobster also use their claws in frequent fights and can be even more aggressive and cause more harm than males, but their claws grow slower, and by the onset of sexual maturation, females trade off enlarged claws with a broader abdomen and egg production (Debuse, Addison, & Reynolds, 1999; Skog, 2009b). Sexual selection is therefore likely to favour male claws as a primary male secondary sexual trait (Atema, 1986; Sørdalen et al., 2018), which could also (at least partly) explain the increased natural mortality rate of males compared to females (Moland et al., 2013). Indeed, injury has been found to be a significant predictor of shell diseases that affect males more than females off the coast of Devon, UK (Davies et al., 2014). Yet, in our study, claw loss seems to be affecting females in MPAs more than females in fished areas, whereas there were no differences in males. This suggests that density and crowding effects might act differently on the sexes, particularly bearing in mind that fighting among females can be more intense (Skog, 2009b) and more frequent when shelters are abundant (Debuse et al., 2003). Regardless, male lobsters are more catchable than females (Moland et al., 2013) and protection of egg‐bearing females ensures that females experience lower fishing mortality rates than males (Jury, Pugh, Henninger, Carloni, & Watson, 2019). Consequently, fisheries selection on females, and female claw size, might also be weaker.

### 4.2 The potential of MPAs for preserving secondary sexually selected traits

The removal of dominant males is likely to disrupt the hierarchical order and subsequently the mating pattern in clawed lobster. Claw size and body size are strong predictors of male mating success, yet these traits have shown to have little influence on male success in fished areas (Sørdalen et al., 2018). This is likely because the combined effect of lower density and reduced mean and variability in male phenotypes (e.g. claw size, body size, relative claw size) leaves female lobster with a reduced opportunity for mate choice. The result of this study, combined with previous findings of strong sexual selection and ongoing fishery selection, suggests the likely existence of an inverse relationship between fishing mortality and sexual selection on male relative claw size in European lobster. When intensive fishing shifts the distribution towards small‐clawed males, it also limits the scope for sexual selection to act upon this trait. On the other hand, our results also show that marine protected areas have the capacity to preserve and promote high variation in male characteristics and thereby to strengthen sexual selection.

Theoretically, as the strength of sexual selection increases within the protected areas, the potential for increased reproductive (and genetic) output from large females (mated with large‐clawed males) could counter opposing selection pressures in nearby fished areas. Additionally, spillover by males with attractive phenotypes to harvested areas (where such males are depleted) can strengthen sexual selection through dispersal and gene flow if they are able to reproduce before they are harvested (Baskett & Barnett, 2015; Pelletier et al., 2014). The capacity of MPAs to buffer fisheries‐induced evolution will depend on the amount of interchange between protected and nonprotected areas, which so far has proven difficult to demonstrate (for a review, see Lorenzo, Claudet, & Guidetti, 2016). One study found a tendency for larger female lobsters to spill‐into the protected areas of this study system (Thorbjørnsen et al., 2018). Since the MPAs house higher quality males, that is males of larger size and with larger claws, such patterns could be due to mate attraction.

Insight on movement behaviour of European lobster (Moland, Olsen, Andvord, Knutsen, & Stenseth, 2011; Skerritt, Robertson, Mill, Polunin, & Fitzsimmons, 2015; Thorbjørnsen et al., 2018; Wiig, Moland, Haugen, & Olsen, 2013) indicates that the MPAs in our study system may be too small to cover the lobsters’ full home ranges, which means that any estimated effect of protection on lobster (in these study populations) is likely to be conservative, since exchange of lobsters over fished area–MPA boundaries will counteract differences in selection pressures. Larger MPAs, big enough to encompass the full home ranges of most inhabiting lobsters, should to a greater extent be able to maintain higher variability in all traits. Although large MPAs may be the silver bullet for restoring natural selection pressures in exploited populations, the rate of establishment of protected areas is slow in most countries. Therefore, traditional fishing regulations might also be better adapted in order to avoid negative trait changes. For example, the entrances of traps could be made smaller to reduce the catchability of large‐clawed lobsters, and regulations lowering fishing mortality (e.g., bag limits, trap numbers) would slow harvest selection and help maintain more natural densities and trait distributions.

The replicated study design, using multiple pairs of protected and fished areas, allows the testing of hypotheses which elevate local‐scale findings towards the identification of general trends. While these results apply to one species in one specific system, our demonstration provides a valuable contribution to fisheries conservation science and should apply broadly to all species with some form of sexually selected trait affected by anthropogenic pressure that can benefit from spatial management actions. Such candidate species can be any species with strong mating competition and/or mate choice with sexual dimorphism (i.e., size, morphology or coloration), sex‐changing fishes with dominance hierarchies or nest‐builders with territorial behaviour, many of which are numerous in both temperate and tropical reef ecosystems. A natural next step should be to investigate the responses of other species in the same study system, for example the sexually dimorphic brown crab (sensu Öndes, Kaiser, & Murray, 2017), green crab (sensu Juanes, Lee, Mcknight, & Kellogg, 2008) and others, to test the generality of the findings. It would also be necessary to disentangle the harvest selection and the potential density effect on morphology, using more and perhaps larger MPAs that are less likely to be impacted by fishing. Studies on trait heritability should give us helpful insights into the underlying mechanisms governing claw traits and how genetics versus plasticity can shape claws under high population density. From an evolutionary perspective, high plasticity could be beneficial to slow down genotypic change.

The implications of the results from this study are twofold. First, our study reveals how fisheries‐induced selection against a male sexual character can drive population changes in such traits. Second, it shows that marine protected areas can rescue secondary sexual traits in harvested populations. The prerequisite is that fishing has not effectively eroded the mechanisms driving phenotypic variation in claw size (i.e., genetic diversity). The discrepancy in trait expression between protected and fished areas also serves as a strong warning signal about unintended consequences of selective fishing. MPAs with animals not selected by fishing will exchange individuals and genotypes with surrounding areas and can therefore be effective in curbing undesired phenotypic selection from harvesting (Baskett & Barnett, 2015; Baskett et al., 2005; Dunlop, Baskett, Heino, & Dieckmann, 2009). Fisheries managers have largely focused on abundance, size/age and composition of target species within protected areas, yet monitoring of changes in sexually selected traits can perhaps be an equally good or additional measure of population status. When the phenotypic variance in sexually selected traits increases after harvesting ceases, as we show in this study, it is reasonable to assume that genetic diversity is also maintained (Carr & Reed, 1993; Quinn, Wing, & Botsford, 1993), because it often plays a key role in stabilizing social systems and maintaining sexual selection. Harvesting refuges like marine protected areas, if well designed and managed, should therefore relax or even reverse the effects of harvest selection or curb fisheries‐induced selection with evolutionary consequences (Rowe & Hutchings, 2003; Tenhumberg, Tyre, Pople, & Possingham, 2004).

## CONFLICT OF INTEREST

None declared.

## ETHICAL APPROVAL

The capture–release and tagging of lobster were carried out under the permission of the Norwegian Animal Research Authority (FDU) and the Norwegian Directorate of Fisheries (for sampling inside the MPAs, ref. No. 11/5207). The European lobster is categorized as being of Least Concern in the Norwegian Red List for Species after the new revisions in 2015 (Artsdatabanken, Norge 2015).

## Supporting information

Fig S1‐S2

## Data Availability

Data available from the Dryad Digital Repository: https://doi.org/10.5061/dryad.m37pvmczq
