# Supplementary material for: Marine protected areas rescue a sexually selected trait in European lobster
Source: Evol Appl. 2020 May 22;13(9):2222–33. doi: 10.1111/eva.12992 (PMC7513721; doi:10.1111/eva.12992)
Supplement: Supplementary file 1 — Fig S1‐S2 [file EVA-13-2222-s001.docx]

**Supplementary information: figure S1 and S2**


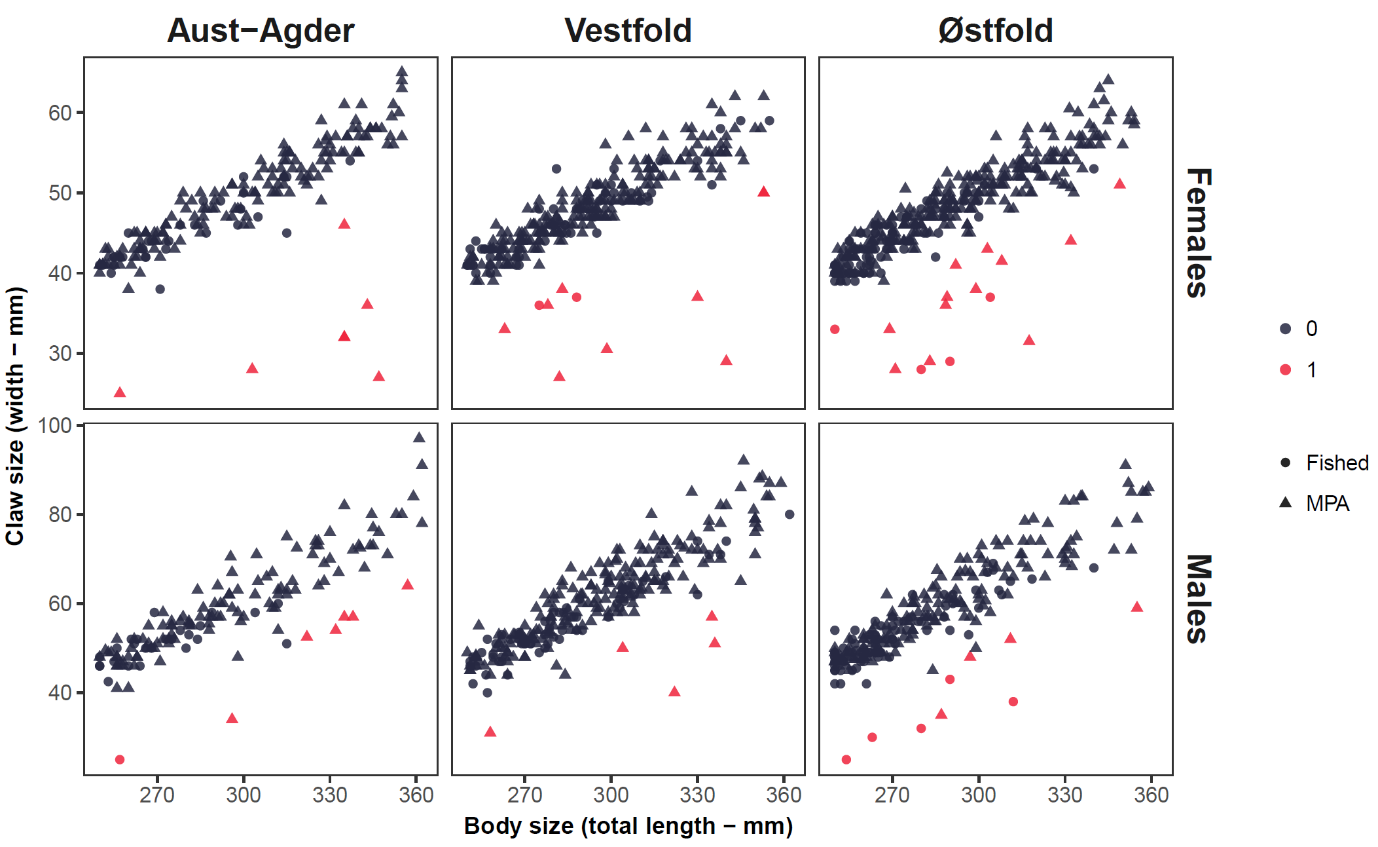


**Figure S1.** Scatterplot of claw width (claw width, *CW*, in mm) and body size (total length, *TL*, in mm) for lobsters (with restricted size-range, see methods) sampled from 2017 – 2019, were red points/triangles indicate those determined to be lobsters with a regenerating claw, using a regression approach; sequentially removing the largest negative outlier (lowest Pearson residual value from the linear models using model structure described in material and methods) and refitting the model until the largest negative outlier was smaller or equal to the largest positive outlier. Lobsters with regenerating claws were not included in the final model (34 females and 21 males). The proportion of lobsters with regenerating claws were 3.83 % in MPAs and 3.35 % in fished areas.

**
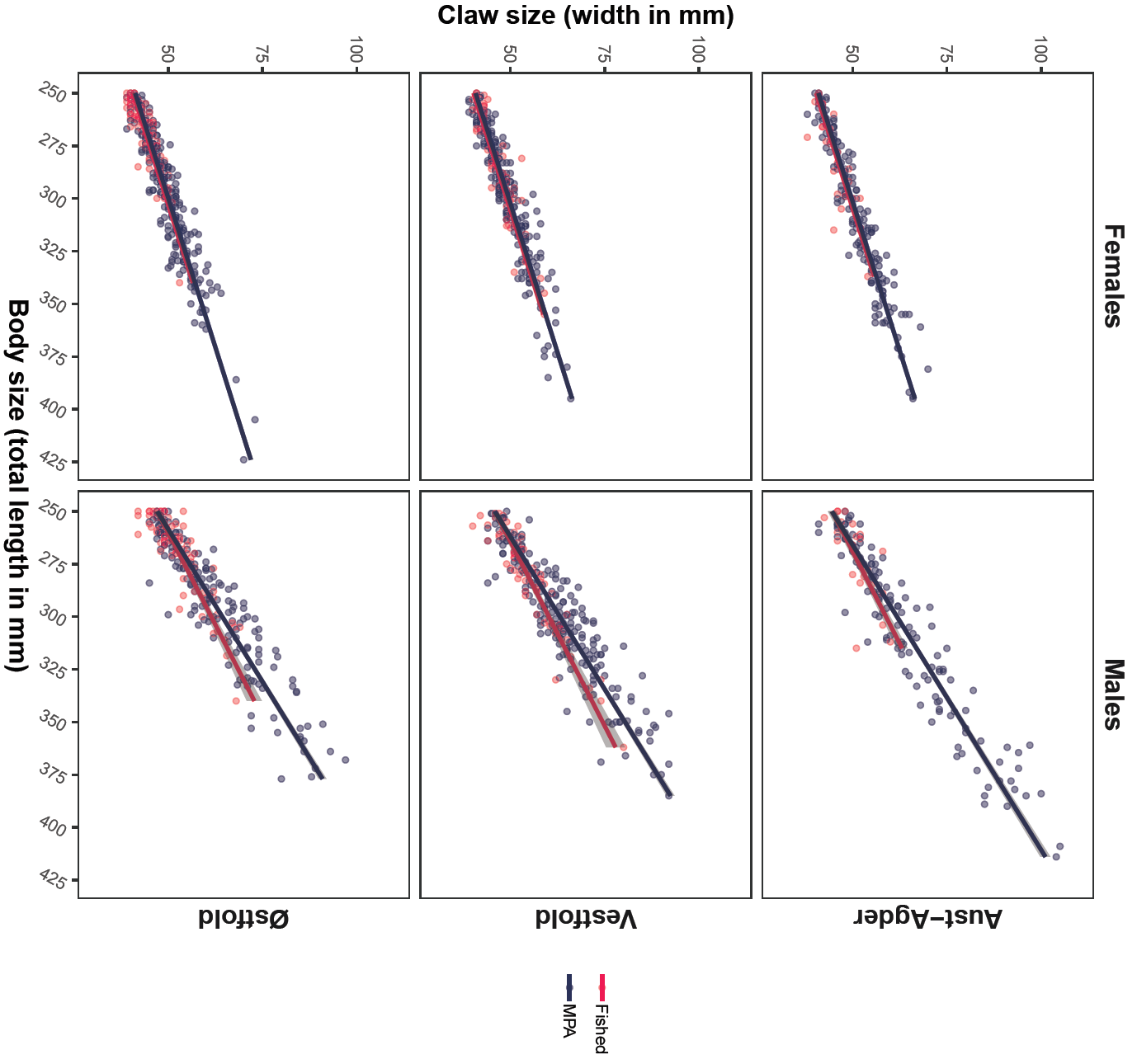
**

**Figure S2.** Un-restricted regression of claw size (claw width, *CW*, in mm) to body size (total length, *TL*, in mm) in female and male European lobster, sampled in lobster MPAs and fished areas of Aust-Agder, Vestfold and Østfold in 2017 – 2019. All individuals above minimum legal-size limit are included. The areas in grey are 95% confidence intervals. Lobsters with regenerating claws (negative outliers) removed; 38 females and 22 males. The models with the highest support were the same models as with the restricted data; the size of claws increased more with increasing body size for males in the MPAs than in the fished area (TL × Status interaction: β=0.06, se=0.02, p=0.00013) and after removing the non-significant interaction effects for females (β=0.007, se=0.007, p=0.351), their relative claw size also differed between fished and protected areas as the additive effect of Status was significant (β=0.58, se=0.17, p=0.0012).
